# Mindfulness in Eating Is Inversely Related to Binge Eating and Mood Disturbances in University Students in Health-Related Disciplines

**DOI:** 10.3390/nu12020396

**Published:** 2020-02-02

**Authors:** Ifigeneia Giannopoulou, Maria Kotopoulea-Nikolaidi, Sofia Daskou, Kathy Martyn, Ashani Patel

**Affiliations:** 1 Senior Lecturer in Sport and Exercise Physiology and Nutrition Sciences University of Brighton, Hillbrow, Denton Road, Eastbourne BN20 7SR, UK; A.Patel26@uni.brighton.ac.uk; 2 Princess Alexandra Hospital, Harlow CM20 1QX, UK; marianikoll@yahoo.com; 3 Nottingham Trent University, Nottingham NG1 4FQ, UK; sofia.daskou@ntu.ac.uk; 4 University of Brighton, School of Health Sciences, Eastbourne BN2 07SR, UK; K.J.Martyn@brighton.ac.uk

**Keywords:** university students, mindful eating, mental health, mood disturbances, binge eating, health-related disciplines

## Abstract

The purpose of the study was to investigate the relationship between mindful eating, disordered eating and mood in university students in health-related disciplines. A total of 221 university students participated in the study; 102 students studied sport and exercise science (SS), 54 students pharmacy sciences (PS), and 65 students health sciences (HS). Participants completed the Binge Eating Scale (BES), the Mindful Eating Questionnaire (MEQ), and the Profile of Mood State questionnaire (POMS). 41% of the students were classified as binge eaters and 57% were above the POMS threshold of depression. Binge eaters were found to have significantly lower MEQ score and significantly higher total mood disturbance scores (TMD) compared to non-binge eaters (*p* < 0.01). Students with a high depression score exhibited no differences in the MEQ score but a significantly higher BES score compared to non-depressed students (*p* < 0.01). Gender differences were found in the MEQ with females exhibiting significantly higher scores in the MEQ score and in all MEQ subscales compared to males, with the exception of the emotional subscale that females were noted to have a lower score compared to males (*p* < 0.01). The MEQ score was inversely related to the BES score (r = −0.30, *p* < 0.01) and TMD (r = −0.21, *p* < 0.05). The MEQ score was a significant negative predictor of the variance of the binge eating behavior of the students (B = −3.17, *p* < 0.001). In conclusion, mindfulness in eating is inversely related to the binge eating behavior and mood state of university students studying health-related subjects and is a significant negative predictor of disordered eating behavior in this high risk population.

## 1. Introduction

Poor mental health in young, university-age adults is becoming a major public health problem [1]. During the last decade, university counselling centers have reported a significant increase in the number of students seeking psychological support, with one in 10 young adults having experienced at least one serious mental health condition [2,3]. Amongst these conditions, eating disorders are considered one of the most prevalent mental health disorders, with adverse health outcomes and with the highest mortality rate among psychiatric disorders [4,5]. In university students, although full syndrome eating disorders are relatively rare, the prevalence of disordered eating behaviors such as binge eating, is greatly elevated in the last decades [6,7]. In particular, binge eating (BE) compared to other disordered eating behaviors, appears to be one of the most frequently diagnosed disordered eating behaviors in university students and if left untreated can lead to bulimia nervosa (BN), binge eating disorder (BED), and anorexia nervosa (AN) [8,9]. Moreover, BE has been shown to negatively affect the mood state of university students by increasing anxiety and depression over food and body image and negatively impacting cognitive state as evidenced by lack of concentration, irritability, and increased absenteeism, factors that can lead to reduced academic performance and quality of life [10,11,12,13].

The etiology of the development of mental health disturbances and disordered eating in university students is complex and needs further investigation. Some of the suggested contributing factors are: Genetic, environmental, changes in social network such as relocation from home in a university campus, and academic competitiveness [14,15,16]. Recently, the academic discipline that the student studies has also been reported to potentially affect eating behavior and mood. It has been suggested that the choice of the university courses might be influenced by pre-existing problematic eating behaviors that are undiagnosed in young adults, as is the case with nutrition students [17]. On the other hand, the academic discipline itself might lead to a greater preoccupation and anxiety over food and body image that might affect the future eating behavior of the students [17]. In particular, students majoring in health-related disciplines such as sports science and nutrition, appear to have a higher prevalence of disordered eating [18,19,20,21]. However, the findings of the literature are still limited and inconsistent, with some studies demonstrating lower or no difference in disordered eating in students of different academic disciplines [8,22,23]. More research is needed to identify whether specific health-related academic disciplines, that put a major focus on health, weight loss, exercise, and sport participation, affect the students’ relationship with food and their body [24]. This is of even greater importance due to the latest popularity and pre-occupation of the young generations with ‘healthy’ and ‘clean diets’ and the increasing stigma of overweight and obesity [25].

Psychological and eating behavior counselling are considered the core of disordered eating treatment, leading to permanent changes in eating behavior [26,27]. Nonetheless, psychotropic medication prescription is still the most commonly used treatment, with a number of adverse side effects [28,29]. Recently, mindfulness and mindful eating have emerged as novel scientific approaches to eating behavior change [30,31,32,33]. One of the first mindful eating interventions, the ‘mindfulness-based eating awareness training’ (MB-EAT) was designed specifically for the treatment of BE with significant improvements observed in eating behavior and binge episodes [34]. Since then, mindfulness-based interventions have been increasingly applied to treating eating-related problems ranging from eating disorders to obesity and its comorbidities [30,31,35,36,37]. However, there is a paucity of data on mindful eating and its relationship to disordered eating and mood in university students [38,39,40]. In a limited number of studies, it has been shown that there is a significant negative relationship between mindful eating and body mass index (BMI) in young adults [38,39,40]. Taylor et al. (2015) has also demonstrated that self-compassion, a central component of the practice of mindful eating, predicts higher mindful eating levels, lower BMI, and lower disordered eating prevalence in students [41]. Moreover, Hendrickson and Ramsmussen (2017) have recently shown that participants in mindful-eating interventions exhibit more self-controlled choices for food [42]. Finally, Arch et al. (2016) have reported significant reduction in portion sizes and a healthier selection of snacks in university students after the introduction of a few brief mindfulness instructions prior to eating [43].

The purpose of the present study was to investigate the levels of mindful eating in university students of health-related disciplines and to assess the relationship between mindfulness in eating with disordered eating and mood disturbances in university students of health-related disciplines.

## 2. Materials and Methods

### 2.1. Participants

A total of 221 students from the University of Brighton, UK participated in the study. From this cohort, 102 students were studying sport and exercise science (SS), 54 students pharmacy sciences (PS), and 65 students health sciences (HS) (i.e., nursing, occupation therapy, and physiotherapy). From the total cohort, 84.2% were females and 14.0% were males (Table 1). Students were recruited from online advertisements and posters placed in the university campus. Participants were informed of all the procedures and signed an informed consent form prior to participation in the study. All procedures were approved by the University of Brighton ethics committee and conformed to the standards set by the Declaration of Helsinki.

### 2.2. Experimental Design and Measurements

Participants completed three questionnaires online. The Profile of Mood states questionnaire (POMS), the Mindful Eating Questionnaire (MEQ), and the Binge Eating Scale (BES) questionnaire.

*Profile of Mood States Questionnaire.* The POMS questionnaire consists of 65 items assessing the mood of the individual. A total mood disturbance (TMD) score is calculated by summing the totals for the five negative subscales, which are: Tension, depression, fatigue, confusion, and anger and then subtracting the positive subscale of vigor [44]. The POMS total score ranges from 0–60. High scores for tension, depression, anger, fatigue, confusion, and TMD reflect a negative mood state, and high scores of vigor reflect a positive mood state. A cut-off point of ≥7 is used in the POMS depression subscale to identify individuals with depression [44].

*Mindful Eating Questionnaire.* The MEQ consists of 28 items and five subscales: Disinhibition, which assesses the individual’s ability to avoid eating when full; awareness, which assesses the individual’s awareness of texture, smell, and taste of food; external cues, which assesses the individual’s inclination to eat in response to external cues; emotional response, which assesses the individual’s tendency to eat in response to negative emotions; and distraction, which assesses the individual’s level of distraction while eating [45]. A MEQ total score and subscale scores are calculated [45].

*Binge Eating Scale.* The BES consists of 16-items evaluating the behavioral manifestations, feelings and thoughts regarding a binge episode. The BES total score is determined by adding the individual’s values for the 16 items and it can range between 0–46. A cut-off point of ≥18 is used in the literature to indicate the presence of binge eating, with scores ≤17 considered as non-bingeing, 18–26 moderate bingeing, and >27 severe bingeing [46].

### 2.3. Statistical Analysis

Data were analyzed using SPSS 21.0. Independent t-test were employed to compare the results between genders, depressed versus non-depressed participants and binge eaters versus non-binge eaters. Pearson-product bivariate correlation analysis was used to investigate the relationship between mindful eating, binge eating, and mood state. Multiple regression analysis was used to determine the predictive value of mindful eating and mood on binge eating. Statistical significance was set at *p* < 0.05. Data are reported as mean ± SEM.

## 3. Results

### 3.1. Eating Behavior

The participants’ total MEQ score was 3.11 ± 0.03 (Table 2). The mindful eating subscales of mindful eating ranged between 2.69 and 5.54, with the highest score noted in the mindful eating subscale MEQ external (5.45 ± 0.06).

**Figure 1:**
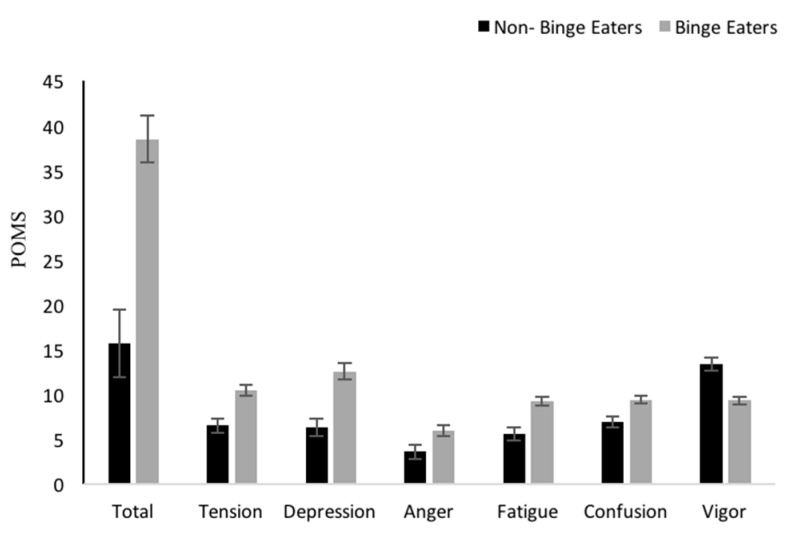

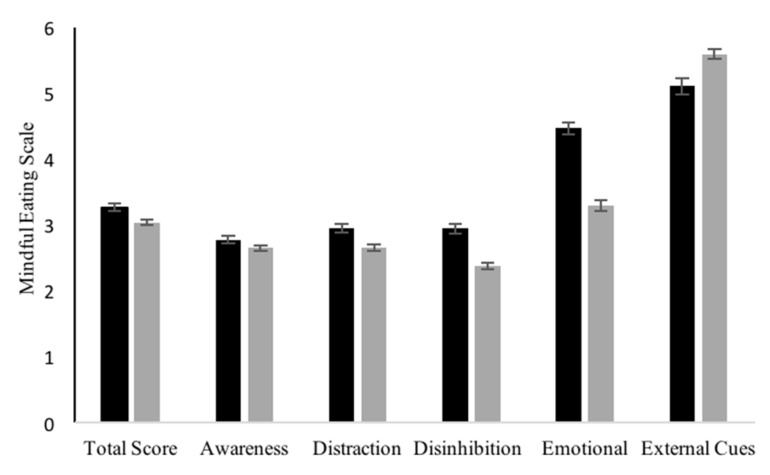
Differences between non-binge eaters and binge eaters in POMS total and subscales and mindful eating total scores and subscales (Mean ± SEM), *p < 0.05. POMS = Profile of Mood State

The BES score for all participants was 13.82 ± 0.52 which classified the students in the non-bingeing category (Table 1). However, from the overall sample of students, 41% of the students were classified as binge eaters [47]. When the students were divided between binge eaters (BE) (*n* = 64) and non-binge eaters (NBE) (*n* = 157), BE were found to have significantly lower total score in mindful eating and lower score in most MEQ subscales compared to NBE (*p* < 0.05). Moreover, BE were found to have a higher score in the MEQ external subscale compared to the NBE (*p* < 0.05). BE had a significantly higher score in total POMS and all POMS subscales (*p* < 0.05) (Figure 1).

### 3.2. Mood State

**Figure 2:**
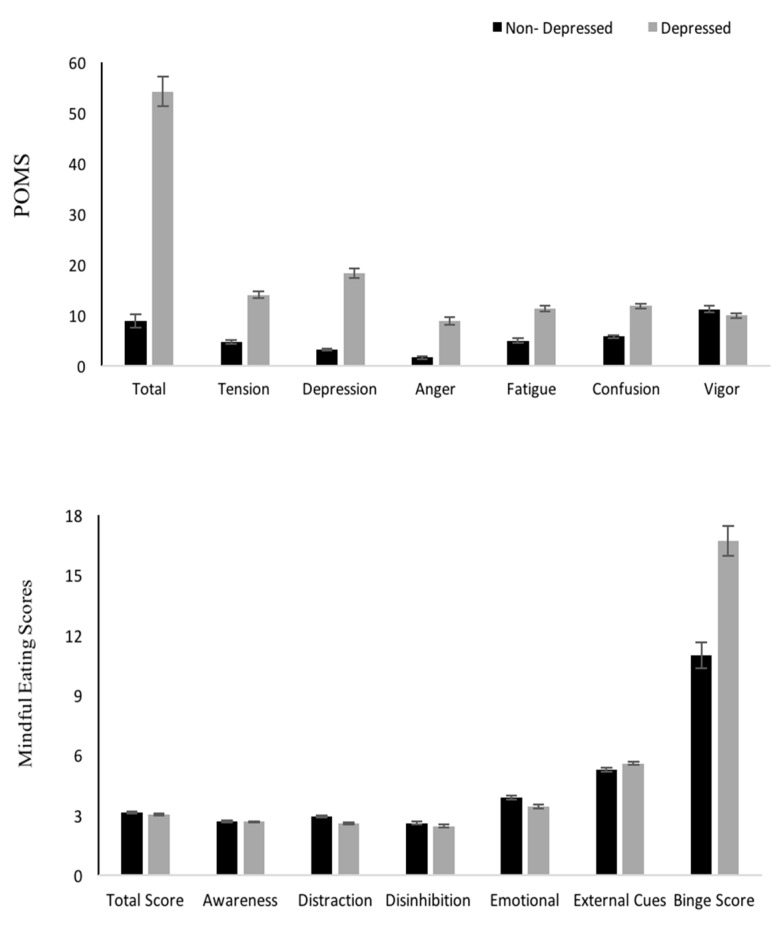
Differences between non-depressed versus depressed students in POMS, mindful total scores and subscales, and total Binge Eating Score (Mean ± SEM). *p < 0.05.

The total POMS score for all participants was 31.93 ± 2.28. From the overall sample of participants, 57% of the university students were above the threshold of seven in the depression score [44]. When the students were divided in groups of depressed (D) and non-depressed (ND), based on the depression threshold, no difference in the total MEQ score was found (*p* > 0.05). However, statistically significant differences were found in MEQ subscales, with D students exhibiting lower scores in the MEQ distraction and emotional subscale and a higher score in the MEQ external subscale compared to ND students. D students had a significantly higher total BES score compared to ND students (Figure 2). The D students exhibited significantly higher scores in tension, anger, fatigue, and confusion compared to ND (*p* < 0.05).

### 3.3. Gender Differences

**Figure 3:**
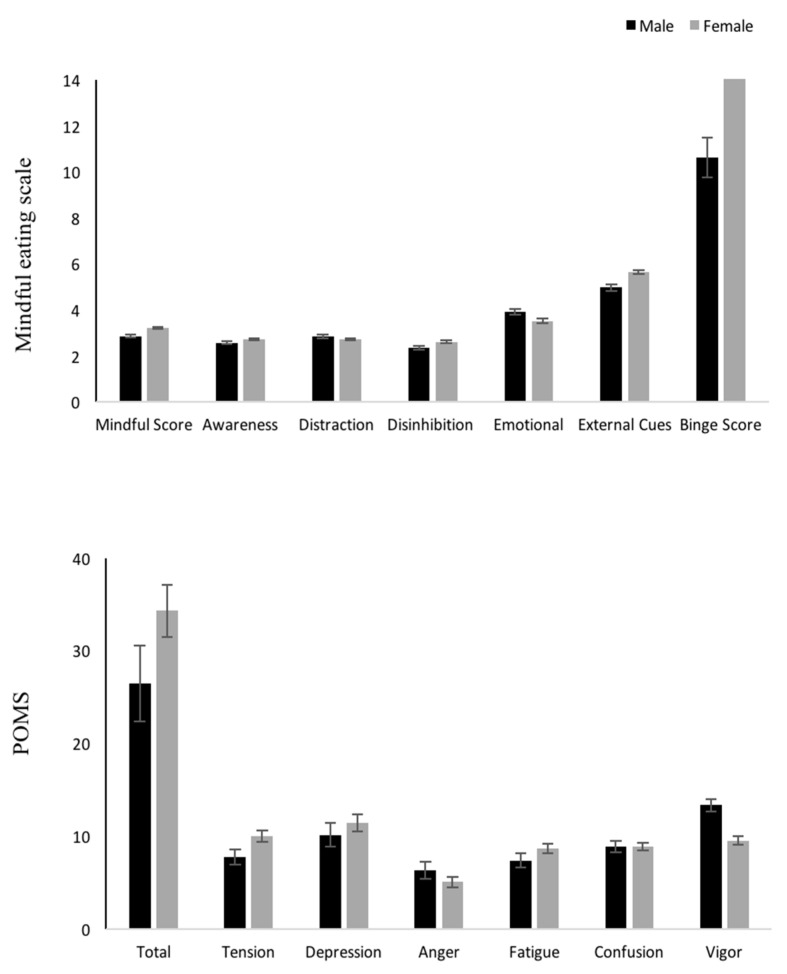
Differences between genders in mindful eating total scores and subscales, binge eating total scores, and POMs total scores and subscales (Mean ±SEM) *p < 0.05.

Females had statistically significantly higher scores in the MEQ total score and in all MEQ subscales compared to males, with the exception of the MEQ emotional subscale that females were noted to have a lower score compared to males (*p* < 0.05). No differences in the MEQ subscale of distraction were found between the two genders (*p* > 0.05). (Figure 3). Females also had a higher score in BES (*p* < 0.05) and in the POMS tension subscale (*p* < 0.05); however, no differences were found in any other POMS subscales and TMD between genders (*p* > 0.05). Males had a higher POMS vigor score compared to females (*p* < 0.05).

### 3.4. Correlations

Significant correlations were found between mindful eating, binge eating, and POMS. In particular, the MEQ total score was inversely related to the BES score (r = −0.30, *p* < 0.01) and TMD (r = −0.21, *p* < 0.05). Significant negative correlations were also found between the MEQ subscales and the BES total score, TMD, and POMS subscales of depression, tension, fatigue, confusion, and anger (Table 3).

A multiple regression analysis was employed to investigate whether mindful eating and TMD could significantly predict the students’ binge eating behavior scores. The regression model was a significant predictor of the binge eating behavior of the students, explaining 28.1% of the variance in BES scores (F(2, 218) = 42.58, *p* < 0.001). Specifically, the MEQ total score negatively predicted the binge eating behavior (B = −3.17, *p* < 0.001) while the TMD positively predicted the binge eating score (B = 0.10, *p* < 0.001) of the students.

## 4. Discussion

A high prevalence of mental health and disordered eating behaviors have been noted in university students worldwide. Mindful eating has been recognized as an effective intervention for disordered eating and related mood disturbances, however, there is paucity of data on mindful eating in university students and its relationship to disordered eating behaviors and mood. The purpose of this study was to investigate the level of mindful eating in university students of health-related disciplines and explore its relationship with binge eating and mood disturbances. We found that the mindful eating level of university students is inversely related to their binge eating behavior and mood. Moreover, the level of mindfulness in eating is a significant negative predictor of the binge eating behavior of the students. Finally, students that exhibit more severe binge eating behaviors were found to have a lower level of mindfulness in eating and more severe mood disturbances, demonstrating the need for effective eating behavior treatment plans for this high-risk population.

This is one of the first studies to the authors’ knowledge that have investigated the level of mindful eating in university students of health-related academic disciplines. We found that overall the students’ mindful eating level is comparable to that observed in previous studies in adult populations [31,37]. However, gender differences were identified in the level of mindfulness in eating in our university student population. In particular, females exhibited more mindful eating behaviors in almost all subscales compared to their male counterparts, with the exception of the emotional subscale, where females were found to be less mindful compared to males. There are no studies to the author’s knowledge investigating the role of gender in mindful eating in university students. It is possible that female students are more mindful in most aspects of their eating compared to males, due to the higher level of awareness, education, and knowledge in nutrition and dieting observed in adult females compared to males [31,37]. The lower level of mindfulness in the emotional subscale found in females in our study, might provide an explanation for the higher scores in binge eating observed in our female cohort. Emotional eating is an important etiological factor of binge eating in females that has been previously reported in the literature [7,12]. More research is needed to further investigate the potential gender differences in mindful eating and emotional eating in university students and their implications in disordered eating, mental health, and well-being.

The level of mindful eating noted in our student population was found to be a significant negative predictor of their binge eating behavior. Specifically, the more mindful the students were in their eating, the lower the score of binge eating and total mood disturbances was. Moreover, the higher the level of mindfulness in the distraction and emotional subscales of mindful eating, the lower the binge eating behavior and depression noted in students. High scores in the distraction and emotional subscales of the MEQ demonstrate the ability of the individual to focus on their food while eating without being influenced by distractions and the ability to resist to emotional triggers of overeating, respectively [45]. Increased mindfulness in these subscales can lead to improvements in the frequency and magnitude of binge eating episodes and related mood disturbances [33]. Our findings are in agreement and add to the limited research evidence in young adults and university students, where mindfulness in eating has been associated with lower disordered eating prevalence and lower BMI [38,41]. Future research studies should further explore this phenomenon and investigate the effects of mindful eating-based interventions on the binge eating behavior and mood of university students.

In the present study, 41% of our student population was classified as binge eaters. Our findings demonstrate the magnitude of the problem of disordered eating in this vulnerable population of young adults. A number of factors have been proposed as contributing to the development of disordered eating in university students such as relocation from home, new social network and eating environment, high academy pressure, and academic discipline [14,15]. Specifically, academic discipline appears to play a significant role in the risk of disordered eating and the development of clinical eating disorders. Previous studies in health-related academic disciplines such as nutrition, dietetics, and sport science have shown that these students exhibit more disordered eating behaviors compared to a control population of students [19,20,24]. More research is needed to identify the possible reasons that students in health-related disciplines might be at a higher risk of developing disordered eating. It is possible that the preoccupation of this student population with health and fitness as well as the recent popularity of ‘clean’ or ‘healthy” diets, create a ‘nurturing’ environment for disordered eating.

Fifty-seven percent of our university student cohort was found to be above the threshold of depression, demonstrating the high prevalence of mental health issues in this young population. Our results agree with recent findings of a high prevalence of mood disorders such as depression and anxiety in university students, significantly increasing the risk of mental health and disordered eating development in the future [48,49,50,51]. This phenomenon is further supported by our finding that mood disturbance is a significant predictor of the binge eating behavior of the students. In addition, it evidenced in the higher binge eating score and lower mindful eating score of our students that were classified as depressed. Our findings are in agreement with the limited existing evidence on the relationship between mood and disordered eating in university students [51] and demonstrate the important role that mood state plays on the development of disordered eating in this population. These findings raise the awareness of the mental health issues that university students face and the high risk for the development of depression. Preventive behavioral treatment plans with the inclusion of mindful eating interventions should be investigated in the university setting, taking into account and focusing on the specific social, psychological, and environmental triggers of disordered eating and mood disturbances that this population is exposed to.

There are some limitations in the present study that must be considered when interpreting the data. Firstly, the present investigation is a cross-sectional study that cannot prove causation but only explore relationships amongst the measured variables. Hence, caution is needed in the interpretation and conclusions of the findings. Secondly, the study’s measures of self-reported data on mindfulness, eating behavior, and mood might not be fully reflecting the actual state of the participants. Lastly, the current sample largely consisted of female participants. It is possible that a more gender-balanced sample could have enriched the results of the study.

## 5. Conclusions

This study has demonstrated for the first time in the literature that mindfulness in eating is inversely related to the binge eating behavior and mood disturbances observed in university students of health-related disciplines and that the poorer the mental health of the students, the lower the level of mindfulness and the more disordered the eating behavior is. In addition, mindfulness in eating has been identified as a significant negative predictor of the disordered eating behavior of university students, exhibiting the potential important role that mindfulness and awareness of eating behavior could have in the treatment of disordered eating and related mood disturbances in this high risk population of young adults. These findings suggest the need for more effective support of the mental health and disordered eating issues that students face in their university years, with a focus on the specific social, psychological, and environmental predisposing factors and triggers of disordered eating that the students are exposed to in universities. Future studies should focus on a more in-depth exploration of the phenomenon of mindful eating in university students and its predictive value in the development of disordered eating and mood disturbances in university students of different academic disciplines. Moreover, future studies should explore how mindfulness in all stages of food consumption, rather than just the eating stage, may bare impact on disordered eating and mood disturbances in university students. Such an exploration will lead to a more comprehensive assessment and understanding of the phenomenon of mindfulness in eating behavior and its effects on the mood state of the vulnerable population of university students. As we move to a more personalized medicine approach, there is a need to personalize and individualize the mental health support and wellbeing of our young adult population of university students.

## Figures and Tables

**Table 1:** Participants’ characteristics, binge eating, and Profile of Mood State questionnaire (POMS) total scores and subscales.

Variables	Student Sample (*n* = 221)
Participants	221
Age, *y*	22.48 ± 0.34
Gender, *(%)*	15.84% Males 84.16% Females
Binge Total Score	13.84 ± 0.52
POMs Total Score	31.93 ± 2.28
POMs Tension	9.38 ± 0.51
POMs Depression	10.81 ± 0.74
POMs Anger	5.30 ± 0.48
POMs Fatigue	8.21 ± 0.42
POMs Confusion	8.75 ± 0.34
POMs Vigor	10.53 ± 0.40

Values are means ± SEM, POMs = Profile of Mood State.

**Table 2:** Mindful Eating Questionnaire (MEQ) total score and subscales for university students.

Variables	Student Sample (*n* = 221)
MEQ Total Score	3.11 ± 0.03
MEQ Awareness	2.69 ± 0.03
MEQ Distraction	2.74 ± 0.04
MEQ Disinhibition	2.54 ± 0.04
MEQ Emotional	3.64 ± 0.07
MEQ External	5.45 ± 0.06

Values are means ± SEM. MEQ = Mindful Eating Questionnaire.

**Table 3:** Correlations between MEQ, binge eating, and mood state (POMS).

MEQ Scale	Binge Eating Total	POMS Total	Depression	Tension	Anger	Fatigue	Vigor
Total MEQ	−0.30 **	−0.21 **	−0.17 **	−0.17 *	−0.20 **	−0.19 **	0.02
Distraction	−0.39 **	−0.35 **	−0.33 **	−0.35 **	−0.20 **	−0.31 **	0.11
Emotional	−0.65 **	−0.32 **	−0.27 **	−0.19 **	−0.22 **	−0.33 **	0.29 **
External	0.22 **	−0.12	0.08	0.13 *	0.02	0.18 **	−0.08
Disinhibition	−0.48 **	−0.14 **	−0.13	−0.06	−0.12	−0.11	0.10
Awareness	−0.12	−0.05	0.02	−0.02	0.04	−0.04	−0.20 **

* Correlation significant at *p* < 0.01; ** Correlation significant at *p* < 0.05.
